# Carbon-based smart nanomaterials in biomedicine and neuroengineering

**DOI:** 10.3762/bjnano.5.196

**Published:** 2014-10-23

**Authors:** Antonina M Monaco, Michele Giugliano

**Affiliations:** 1 Theoretical Neurobiology and Neuroengineering Lab, Department of Biomedical Sciences, University of Antwerp, Universiteitsplein 1, B-2610 Wilrijk, Belgium; 2 Brain Mind Institute, Swiss Federal Institute of Technology, Lausanne, CH-1015, Switzerland; 3 Department of Computer Science, University of Sheffield, S1 4DP Sheffield, UK

**Keywords:** carbon nanotubes, electrophysiology, graphene, microelectrodes, nanodiamonds, nanotechnology, neuroengineering, neuronal cultures, neuroscience

## Abstract

The search for advanced biomimetic materials that are capable of offering a scaffold for biological tissues during regeneration or of electrically connecting artificial devices with cellular structures to restore damaged brain functions is at the forefront of interdisciplinary research in materials science. Bioactive nanoparticles for drug delivery, substrates for nerve regeneration and active guidance, as well as supramolecular architectures mimicking the extracellular environment to reduce inflammatory responses in brain implants, are within reach thanks to the advancements in nanotechnology. In particular, carbon-based nanostructured materials, such as graphene, carbon nanotubes (CNTs) and nanodiamonds (NDs), have demonstrated to be highly promising materials for designing and fabricating nanoelectrodes and substrates for cell growth, by virtue of their peerless optical, electrical, thermal, and mechanical properties. In this review we discuss the state-of-the-art in the applications of nanomaterials in biological and biomedical fields, with a particular emphasis on neuroengineering.

## Introduction

Over the past few years, the gap between materials sciences and biology has increasingly narrowed. This has enabled substantial progress within interdisciplinary approaches, particularly in those combining micro- and nanotechnologies in biological and biomedical applications. For example, the field of neuroengineering was established, as a recent new research discipline within the field of neuroscience. Neuroengineering focuses on the development of artificial devices and novel materials to be functionally and structurally interfaced with the central nervous system (CNS). Among its ultimate goals, repairing, replacing, and enhancing the function of damaged brain tissue is a priority, as witnessed by the recent progress in (pre)clinical neuroprosthetics and brain pacemakers. At the same time, neuroengineering also deals with serious clinical challenges, due to the unique anatomy and physiology of the CNS, compared to other organs. Today, there is the expectation that materials science and nanotechnology will be able to address these challenges and lead to breakthroughs at the level of the interfaces between artificial transducers/actuators and living cells.

Nowadays, fundamental research in neuroengineering aims to open up new frontiers in tissue engineering through reconstructive/repairing strategies that will ultimately be able to provide a functional bridge to the damaged tissue and restore functions via implantable assisting devices. This calls for the use of new smart materials, whose interactions with living tissue can be controlled, engineered and modified. Nanomaterials are ideal candidates for such applications and by virtue of their nanoscale dimensions, share with biological (sub)cellular structures a similar level of organisation. This review presents some of the recent applications of nanomaterials that were reported in very recent years with an emphasis on carbon-based materials.

## Review

### Carbon and its nanoderivatives: chemical and physical properties

In nature, carbon is found in different forms or *allotropes*, depending on its chemical valence. The electron configuration of carbon in its fundamental state (1s^2^ 2s^2^ 2p^2^) is such that it can form, at the most, two covalent bonds. However, in its chemical compounds, carbon is able to form up to four covalent bonds thanks to a rearrangement of its electron configuration: One of its 2s electrons “moves” into the empty 2p orbital, giving rise to four bonds due to four hybrid orbitals. The three possible hybridisations (sp, sp^2^ and sp^3^) differ according to the number of p orbitals mixed with the s orbital, and according to the angle between the orbitals themselves. This angle, in turn, defines the type of chemical bond carbon can establish; these consist of only simple bonds (σ type) in sp^3^ hybridisation, double bonds (one σ and one π) in sp^2^, and triple bonds (one σ and two π) in sp.

Among the existing allotropes, the most widely known are carbon nanotubes (CNTs) and fullerenes, graphite and graphene (sp^2^), and diamond (sp^3^). From these distinct hybridisations, different properties are inherent to these allotropes.

**Carbon nanotubes (CNTs):** CNTs, first reported by Iijima in 1991 [1], are hollow cylinders made of one (i.e., single-walled CNTs, SWCNTs) or several (i.e., multi-walled CNTs, MWCNTs) layers of graphene. They are obtained by a variety of methods, including chemical vapour deposition (CVD) and arc-discharge, and their electronic properties depend solely on geometric parameters, such as diameter and chiral angle. These parameters are in turn determined by the magnitude and the direction of the chiral vector [2], and their influence is clearly expressed in the electrical conductivity of the CNTs: SWCNTs can be either metallic or semiconducting [3], while MWCNTs show only metallic behaviour [4]. Moreover, the mechanical and electronic properties of SWCNTs can be altered by means of external electric fields [5–6].

The chemical bonds between carbon atoms in CNTs are among the strongest known and this, combined with their characteristic tubular structure, endows CNTs with an extremely high mechanical strength [7], while at the same time exhibiting a very low weight. Combined with a large surface area, these electronic and mechanical properties give CNTs a great potential for microelectronics and optics, and also for biomedical applications (e.g., as nanoelectrodes for neural stimulation and functional scaffolds for tissue engineering).

**Nanodiamonds (NDs):** As a result of the complete sp^3^ hybridisation of its carbon atoms and its characteristic tetrahedral configuration, diamond shows interesting and peculiar properties such as an extreme hardness, low friction coefficient, high mobility of electrical charge carriers and high thermal conductivity [8–9]. Diamond exhibits these properties both in bulk as well as at the nanoscale and combines them with typical features of nanomaterials, such as a large surface area and small overall size.

NDs, mainly obtained by detonation of TNT-like explosives under oxygen-deprived conditions, are core–shell like particles with a diameter of 2–8 nm. Their structure consists of an sp^3^-hybridised core surrounded by layers of amorphous and sp^2^-hybridised carbon [10–12], and they show a strong tendency to agglomerate [13] in clusters with sizes ranging from 100–200 nm to 20–30 μm. This tendency is likely the consequence of several surface terminations, all containing oxygen functional groups such as carboxyl, hydroxyl, ketone and lactone. Ad hoc surface functionalisation of NDs is thus essential for improving the solubility of NDs and to make them suitable for biological applications [14].

The optical properties of NDs are remarkable: highly pure diamond is fully transparent and colourless, whereas the presence of lattice impurities and dopants render diamond coloured [15]. When NDs are excited by UV light [16], they display fluorescence over a broad emission band in the visible region [17] of the spectrum. One of the most common and studied defects in diamond lattice is the *nitrogen-vacancy* centre (NV centre) [18], formed by a lattice vacancy and an adjacent nitrogen atom in the NDs. The importance of these NV centres lies in their magnetic properties [19]: Given the coupling between the spin state and luminescence, the luminescence of NDs can be modulated by local magnetic fields [20–21].

**Graphene:** Graphene is a mono-atomic, two-dimensional, sheet of sp^2^-hybridised carbon atoms arranged as a honeycomb lattice. Since the first single-layer sample was isolated from graphite by Novoselov and colleagues [22] in 2004, graphene has attracted substantial interest and attention for its unique chemical and physical properties, because its existence had for a long time been considered to be impossible [23–24].

Its special electronic structure bestows graphene uncommon and astonishing electronic properties, such as the quantum Hall effect, which can be observed even at room temperature [25], a very high electron mobility [26], the ambipolar electric field effect, the ballistic conduction of electronic charge carriers [27], as well as the nature of the charge carriers themselves, which behave like massless relativistic particles and are thus better described by Dirac’s rather than Schrödinger’s equation [28]. These, and other remarkable properties, including its excellent mechanical strength [29], the high transparency of single-layer graphene [30], and its large surface area [31], make graphene and graphene oxide (GO) one of the most promising materials for technological and biomedical applications.

### Carbon-based nanomaterials in biomedical applications

The peculiar ability of several nanomaterials to functionally integrate with biological systems is a consequence of their interactions with cells and membranes occurring at the subcellular level. However, due to their chemically inert surface and van der Waals forces occurring at the surface, carbon-based nanomaterials, particularly pristine CNTs, tend to agglomerate, which results in a limited dispersion in organic matrices. To overcome this problem and to improve the biocompatibility, or to specify the targeting of the particles, functionalisation methods have been developed and successfully used in the past decade. In the following paragraphs, we review some of the most important biological applications of these nanomaterials.

**CNTs:** The previously mentioned large surface area and their excellent chemical stability confers CNTs the ability to conjugate and absorb several therapeutic molecules, paving the way for using them as drug- and gene-delivery systems. Yang and colleagues [32], for example, exploited the ability of CNTs to cross the blood–brain barrier to deliver acetylcholine into the lysosomes of neurons in the experimental treatment of Alzheimer’s disease in mice.

However, the biological applications of CNTs require their complete purification from both metal and carbonaceous particles [33]. In addition, their surface functionalisation must be designed to enhance their solubility in biological media [34]. In particular, two functionalisation procedures for CNTs are explored in the literature: (i) the non-covalent approach [35–36] that consists of coating CNTs with surfactants, peptides, polymers, or nucleic acids, which preserve their aromatic structure, and (ii) the covalent approach [37–39], by means of applying several protocols, such as oxidation in strong acids, fluorination [40], and Bingel [41] and Billups [42] reactions.

Given the nature of their applications, biocompatibility of CNTs is a crucial, yet still controversial point. How physicochemical characteristics (i.e., length, diameter, and surface functionalisation) affect the toxicity of CNTs [43], and by what mechanisms CNTs can enter the cellular cytoplasm, and where they are localised once internalised [44], remain open questions. In fact, both toxicity and biocompatibility have been reported and discussed extensively for CNTs in recent literature. Inhalation of pristine raw SWCNTs has been described to result in changes in pulmonary functions, inflammatory reactions, and the formation of granulomas [45]. Granulomas and inflammatory reactions have also been reported upon injection of CNTs in the peritoneal cavity, likely as a consequence of their asbestos-like structure [46]. The toxic effects of raw CNTs have also been reported in vitro [47–50], interpreted as a likely consequence of their hydrophobic surface and as a result, their tendency to aggregate*.* However, these adverse effects appear to be reduced for functionalised CNTs [49–50]. Therefore, the cyto- and genotoxicity of CNTs appear to be sample-specific, and require the evaluation of biocompatibility properties on a case-by-case basis.

Despite the debate on their biocompatibility when in solution, CNTs have been proposed as an ideal material over quite a wide range of biomedical applications; in addition to the discussed drug [51] and gene [52–53] delivery, CNTs have been used as biosensors [54], in hyperthermia therapy for tumours [55–56], in tissue engineering [57], for in vivo [58] and in vitro [59] imaging.

The electrical conductivity of CNTs lies at the foundation of the proposal for employing CNTs as smart-scaffolds for excitable cells such as neurons [60] and cardiac cells [61], within regenerative applications. Martinelli et al. [62–63] cultured neonatal rat ventricular myocytes on CNTs substrates and measured the active and passive membrane electrical properties of both single myocytes and multinucleated cells by patch-clamp cellular electrophysiology. In both cases, cells grown on CNTs substrates showed a more negative resting potential compared to the control condition, while no significant differences were found for input resistance and cell capacitance or for the occurrence frequency and kinetics of action potentials (APs). However, this study further highlighted that interfacing cardiomyocytes with CNTs accelerates cells maturation, resulting in an increased expression of mature phenotype-related genes.

Lin and colleagues [64] studied in vitro how pristine SWCNTs dispersed in an extracellular medium can affect the viability of vascular adventitial fibroblasts and their transformation into myofibroblasts. Their results showed an up-regulated expression of a specific differentiation marker, accompanied, however, by an increased generation of the most biologically significant free radicals, the reactive oxygen species (ROS).

**NDs:** Among the applications of NDs, the most important include drug delivery [65–66], implants coating [67] and bioimaging [68]. Similarly to the use of CNTs, the first consideration in biological applications is the biocompatibility of NDs. Diamond, in its bulk form, is chemically inert. However, because surface chemistry is predominant at the nanoscale compared to that of the bulk scale, investigating ND biocompatibility has been a priority in recent years.

One of the first studies in this area was conducted by Yu and colleagues [69], who evaluated the cytotoxicity of fluorescent NDs by employing human embryonic kidney cells: they observed that NDs slightly affected cell viability, even for a concentration of about 400 μg/mL.

Schrand and co-workers [70–72] extensively studied this issue through standard in vitro cell viability assays (i.e., MTT) and also monitored adenosine triphosphate (ATP) production and ROS generation. They found that, compared to several carbon-based alternative nanomaterials (i.e., carbon black, SWCNTs and MWCNTs), NDs were the least toxic when exposed to neuroblastoma cells, as they did not induce significant ROS production and did not affect mitochondrial membrane integrity.

A different approach to biocompatibility of NDs has been proposed by several research groups, who monitored gene expression of cells. No significant change in the expression of Bcl-x and TNF-α genes [65] was found, while a decreased expression of genes responding to genotoxic compounds was described; in addition, no effects on the expression of genes responding to oxidative stress were observed [73]. However, Xing et al. [74] observed that embryonic stem cells responded to incubation with NDs with an increased expression of MOGG-1 and P53, which are proteins related to DNA repair processes. This genotoxicity was increased when cells were incubated with oxidised NDs, suggesting it was a specific consequence of the surface chemistry of NDs. Nonetheless, Xing and co-workers noted that NDs and oxidised NDs induce overall less DNA damage than that caused by MWCNTs.

The investigation of the cellular uptake mechanisms of NDs is also a key aspect for biological applications of NDs. Vaijayanthimala and colleagues [75] reported that cellular uptake was strictly related to the surface functionalisation of NDs and that it took place through clathrin-mediated, energy-dependent, endocytosis processes. Schrand et al. [76] also investigated the uptake of fluorophore-conjugated NDs by neuroblastoma cell line and observed that the fluorophore-conjugation was not affected by the different pH conditions encountered during the uptake process. In addition, NDs were described as localising mainly in endosomes, lysosome and in some cases, the cytoplasm.

Given the possibility for NDs in powder form to spread in the air during detonation synthesis, an in vivo evaluation of their toxicity also became relevant and timely; several studies have focused on this aspect and highlighted that NDs have no remarkable adverse effects in the lungs [77]. In addition, subcutaneous exposure to NDs does not trigger inflammatory responses and NDs do not affect the normal internal organs development or reproductive abilities [78]. In vivo system-level localisation of NDs, studied by labelling NDs with different radionuclides, revealed that NDs preferentially localise in the lungs, liver and spleen [79], and that the urinary system excrete them [80].

Hydrogels and thin-films based on NDs have also been used as drug delivery systems by virtue of their ease of surface functionalisation and small size. They have been reported for successfully delivering several anticancer drugs and preserving their activity under biological conditions [81–84].

The affinity of NDs for protein adsorption has been finally utilised to separate recombinant proteins from *Escherichia coli* [85], resulting not only in a radically faster process than the commonly used purification treatments, but also in a high degree of purity of the recovered proteins, which can be further analysed by mass spectrometry [86].

**Graphene:** Graphene, graphene oxide (GO) and reduced graphene oxide (r-GO) have been investigated as new biocompatible material by virtue of their unique properties, making them suitable for a broad variety of applications. The biocompatibility of graphene is, however, still disputed, given that very few studies are available and because several graphene forms can be employed (i.e., single or few layer, nanosheets, GO, r-GO) each with different chemical and physical properties. These differences can induce distinct toxicological responses in biological systems and require a systematic investigation.

In vitro studies, carried out on human cell lines (i.e., HepG2, BEAS-2B, PC12, hMSCs), have demonstrated that the cyto- and genotoxicity of graphene depends on the dose, shape, and size of the nanomaterial itself [87–89], as well as on the presence of metal contaminants and the residues of the GO preparation method in graphene samples [90].

Biomedical applications of graphene and its derivatives range from photothermal tumour ablation therapy to biosensors, from gene therapy [91] and drug delivery to substrates for biomolecular imaging [92–93], and from tissue scaffolds [94] to electrodes for neural stimulation [95].

For instance, Yang and colleagues [96] investigated the possibility of using polyethylene glycol (PEG)-coated nanographene sheets showing absorption in the near-infrared region as a photothermal agent for in vivo cancer treatment, while scrutinising the effects originating from different graphene sizes and coatings [97].

Several research groups have focused on graphene as biosensors. Dey et al. [98] developed an amperometric cholesterol biosensor; Tang and co-workers [99] studied the electrochemical behaviour of reduced graphene sheet films as nicotinamide adenine dinucleotide (NADH) sensors, while Kim and colleagues [100] and Sun and colleagues [101] studied graphene/Pt-based sensors for the detection of dopamine, and uric and ascorbic acids.

The presence of reactive functional groups and of localised π*-*electrons, which promote the π–π bond with aromatic compounds, render graphene a suitable candidate as a drug delivery system. Several remarkable studies in this area have been performed, including those involving the functionalisation of GO by PEG and water-insoluble chemotherapeutic drugs [102], as well as research involving sulfonic acid groups bound with folic acid in order to target human breast cancer [103]. Furthermore, Weaver and co-workers [104], exploiting the conductive properties of GO sheets, developed an electrically-controlled system capable of a linear release profile of an anti-inflammatory drug.

### Carbon-based nanomaterials in neuroscience

The (sub)cellular organisation of the CNS and carbon-based nanomaterials share some intriguing similarities. This has inspired several research groups to explore the use of these nanomaterials for developing nanosized sensing/actuating technologies, ultimately capable of functional interfacing with nerve cells and brain tissue, in order to repair the brain on the (sub)cellular scale.

**CNTs:** The first application of CNTs in neuroscience was the exploration of CNTs thin-films as ex vivo neuronal growth substrates [105]. Mattson and colleagues reported that MWCNTs favoured neuronal adhesion although neurite branching was reduced, with respect to control conditions. This pioneering work laid the foundations for subsequent studies aimed at establishing the ability of CNTs to support neural adhesion. Since then, several studies [106] have revealed that CNTs are able to guide neuronal adhesion and to impact neuronal networks.

**Microelectrode arrays (MEAs – extracellular recording and stimulation of neuronal activity):** MEAs are devices consisting of metallic microelectrodes (i.e., made of Au, Pt, or titanium nitrate) and embedded in a planar substrate, arranged in an array and connected to an external electrical circuitry. By using individual microelectrodes it is possible to stimulate or record neural electrical activity non-invasively, both in vivo and in vitro. For these applications, which represent the frontiers of neuroprosthetics and brain pacemakers, MEAs should exhibit excellent biocompatibility, large signal-to-noise ratio (S/N), large charge-injection limits, and high spatial resolution. While the latter aspect can be improved by microphotolitography and ultimately designing smaller microelectrodes, reducing the electrode surface exposed to the electrolyte or in close proximity to neuronal cell membranes, has been shown to lead to a significant electrochemical impedance of the interface, decreased injected charge limits and poor S/N properties.

CNTs, by their excellent electrical properties and large surface area, immediately presented themselves as top candidates for the fabrication of a new class of electrodes [107–110]. Gabay and colleagues [111] developed CNTs-MEAs with improved electrochemical properties by synthesizing “islands” of CNTs on silicon dioxide substrates, and confirmed the capability of the device for recording the spontaneous activity of cultured rat cortical neurons.

Gabriel and co-workers [112] deposited a solution of SWCNTs onto conventional platinum electrodes of a MEA, thus enhancing the electrical properties of the device and successfully tested it to record the extracellular activity of ganglion cells in rabbit retinas as a potentially important step for retinal prostheses.

Shein et al. [113] cultured neurons on specifically designed microelectrode arrays in which CNTs had been deposited by CVD on TiN leads, and reported a direct electrical interfacing between neurons and microelectrodes made of CNTs.

Deposition of CNTs onto TiN microelectrode arrays was proposed by means of a micro-contact printing technique by Fuchsberger and colleagues [114], and showed superior recording properties compared to commercial TiN microelectrodes. The same microelectrodes were also employed to electrically stimulate and record neuronal activity, as well as to detect low concentrated amounts of dopamine.

To overcome the problems related to spatial resolution and S/N, more recently Gerwig et al. [115] developed a PEDOT–CNT composite that combined the ionic and electronic conductivity of poly(3,4-ethylenedioxythiophene) (PEDOT), a conductive polymer, with the high mechanical stability of CNTs. This combination, employed as a coating layer of conventional MEAs, resulted in reduced impedance, and thus in improved performances not only when compared with TiN or Au electrodes, but also compared to pure PEDOT electrodes. The interpretation of this phenomenon can most likely be found in the conductivity of the meshwork of CNTs, which enhances the electrical conductivity of the whole composite. Furthermore, PEDOT–CNTs-electrodes demonstrated excellent biocompatibility, allowing for the adhesion of primary chicken cardiomyocytes and the development of a two-dimensional syncytium, accompanied by good quality recording after six and ten days in vitro (DIV).

**Figure 1:**
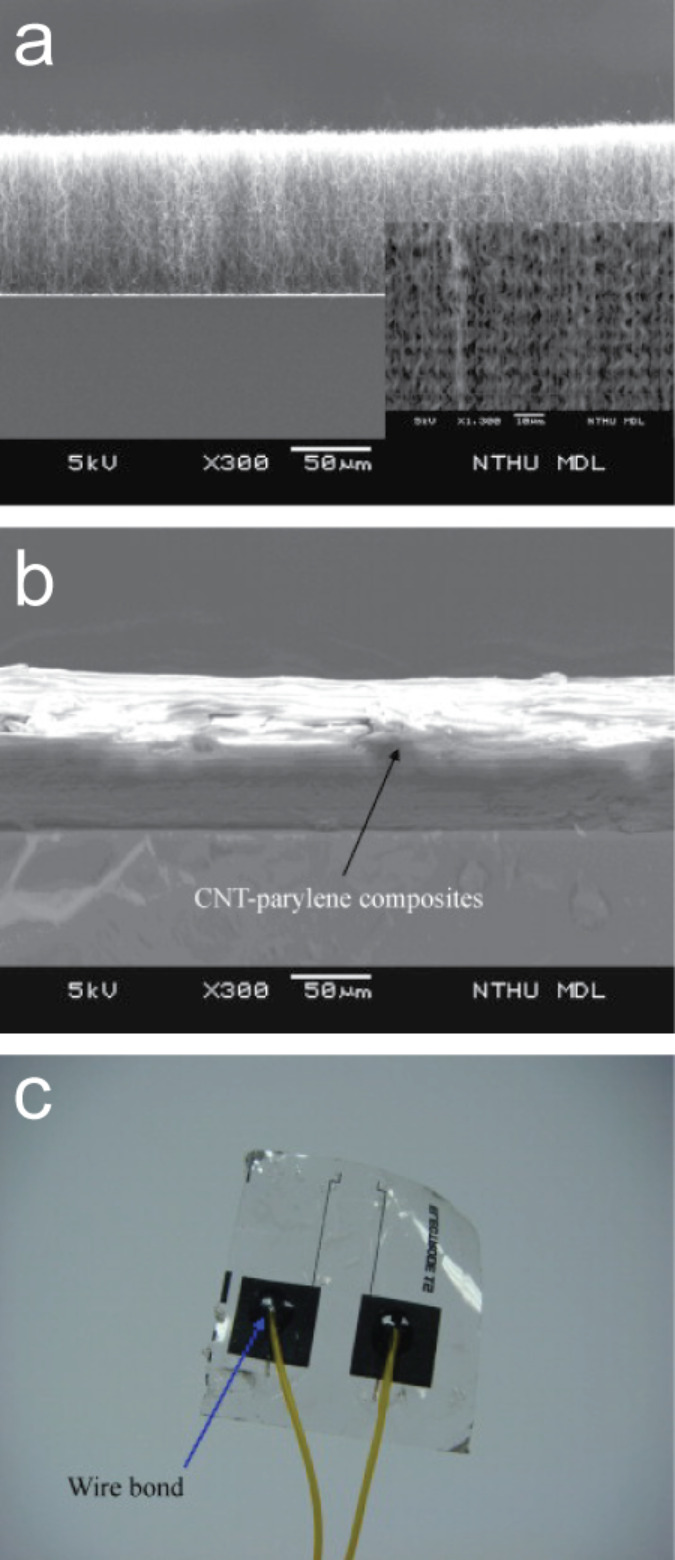
Flexible MEA developed by Lin and colleagues. SEM micrographs showing vertically aligned CNTs on Parylene-C (a,b) and a photo of the transparent flexible CNTs electrodes (c). Reproduced and modified from [116] with permission. Copyright 2009 Elsevier.

The MEAs thus far discussed are designed for in vitro electrophysiological stimulation and recording; however, they are not directly suitable for in vivo applications, in which flexible substrates must be employed. Lin and colleagues [116] were the first researchers to grow CNTs electrode arrays on a silicon substrate, transfer it onto a flexible and biocompatible polymeric film (Figure 1), and then successfully record the APs of crayfish nerve cords.

Another flexible MEA, completely made from CNTs that have been embedded in a polymeric support, has been presented by David-Pur et al. [117]. This device showed the same characteristics as planar CNTs-MEAs, combined with the advantages of being flexible and of consisting of a continuous rough surface, i.e., the CNTs film, which was demonstrated to improve cell adhesion.

**Figure 2:**
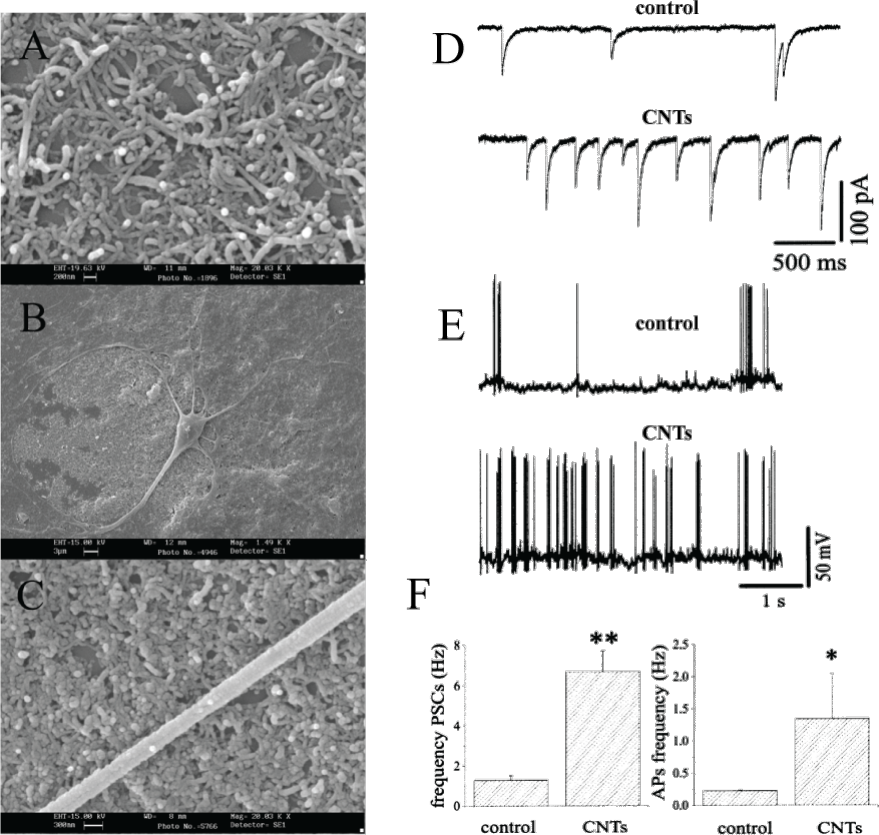
CNTs thin-films are optimal substrates for neuronal growth and development ex vivo (A–C) and improve spontaneous synaptic activity**,** as shown by the increased frequency of (D) post-synaptic currents and of (E) action potentials. Reproduced and modified from [118] with permission. Copyright 2005 American Chemical Society.

**Patch-clamp electrophysiology:** One of the first studies reporting on the electrical activity of in vitro neuronal networks coupled to MWCNT-substrates was that of Lovat and co-workers [118]. They were the first researchers to intracellularly monitor the electrical activity of neurons developing ex vivo on CNT-substrates (Figure 2A–C). The researchers unexpectedly reported that CNTs had an effect on spontaneous synaptic activity (Figure 2D–F).

Mazzatenta and colleagues [119] further characterised and explored this phenomenon by culturing hippocampal neurons on pristine SWCNT-substrates. They also stimulated neurons electrically through the CNTs film and devised a mathematical model for the description of the electrical interface between neurons and CNTs. This work confirmed the improved synaptic activity observed by Lovat et al. [118], though no remarkable differences in cell morphology or neuronal density were found, suggesting that the reason for the enhanced network activity might have resulted from tighter connections between the neuronal membranes and the conductive CNT-substrates.

The presence of these intimate connections was then proved, by means of transmission electron microscopy (TEM), high resolution scanning electron microscopy (HRSEM) and immunofluorescence confocal laser scanning (CLS) microscopy, also conducted by Sorkin et al. [120], who suggested a correlation between mechanical forces and neuronal outgrowth, and by Cellot et al. [121], who reported a very tight yet discontinuous contact between CNTs substrates and neuronal membranes.

**Figure 3:**
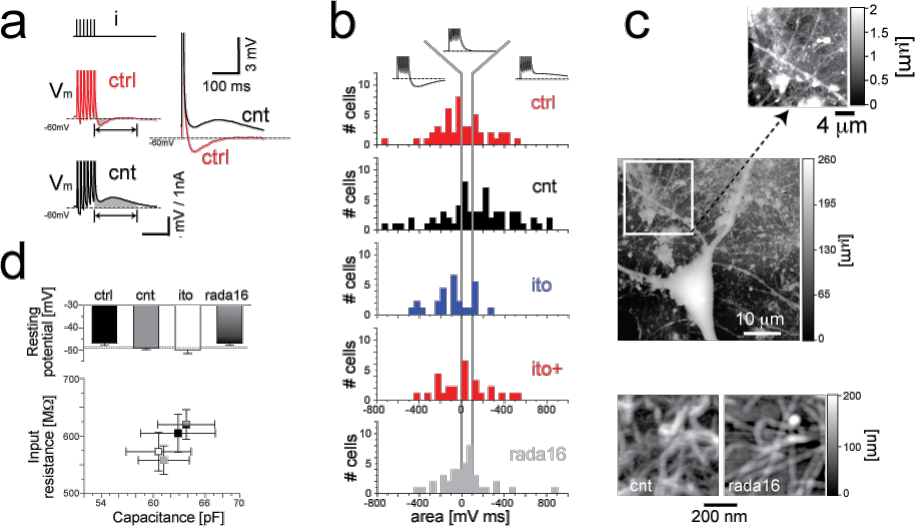
CNTs affect single-neuron excitability, inducing depolarising after-potentials (a). This behaviour is specifically attributable to CNTs, as it has been observed neither for cells grown on smooth and electrically conducting indium tin oxide substrates (ito) nor on electrically-insulating RADA16 peptide thin-films (b), characterised by a similar nanoscale roughness s CNTs (c). The culture substrates do not alter the electrical passive properties of neuronal membranes (d). Reproduced and modified from [121] with permission. Copyright 2009 Nature Publishing Group.

Another remarkable aspect is that the passive properties of the membrane (i.e., quantified in terms of electrical circuit equivalent, as the input resistance, the membrane capacitance and the resting membrane potential) were comparable to the control conditions [118–119,121–122] (Figure 3d). Ultimately, the increase in spontaneous activity, exhibited by neurons in the presence of CNTs, was not a result of an altered cell morphology.

Cellot and colleagues [121] also focused their efforts on the occurrence of an electrical coupling between CNTs and cell membranes. Using patch-clamp electrophysiology, they showed that CNTs strongly impacted the electrical regenerative properties of neuronal membranes upon inducing depolarising after-potentials, which might be related to Ca^2+^-mediated electrogenesis and backpropagating APs (Figure 3a,b). It is worth noting that this behaviour was shown to be specifically attributable to CNTs, as it was not observed in neurons growing on other substrates exhibiting large electrical conductivity or nanoscale roughness (Figure 3c). Moreover, in the same work, mathematical modelling and electrophysiology were used to probe a contingent connection between the effects observed at single-neuron level and those detected at the level of neuronal networks, leading to the speculation that the tight discontinuous connections observed between cell bodies and CNTs might act as shortcuts and transfer electrical currents between different neuronal compartments. Cellot and co-workers [123] also investigated the effects of CNTs at the synaptic level and observed not only that growing neurons on CNT-substrates increased the possibility of finding synaptically-connected pairs of cells, but also that CNTs affected synaptic plasticity.

Another interesting feature of CNT-based substrates is that they are able to modulate the developmental onset of neuronal electrical activity in culture, as reported by Fabbro and colleagues [122]. In their study, the researchers observed that spinal neurons grown on CNTs scaffolds displayed an early developmental onset for generating APs and voltage-gated currents, as well as changes in the expression of genes involved in cell communication, growth, differentiation and in neuronal maturation.

The chronic interface between CNTs and organotypic cultures of embryonic mouse spinal cord and dorsal root ganglia has also been investigated [124]. The researchers observed an improved outgrowth of cellular processes emerging from the dorsal root ganglia, despite having a slightly different morphology than in control conditions. Patch-clamp experiments finally revealed the effect of CNT on spontaneous electrical activity of spinal networks (i.e., an increased amplitude of spontaneous postsynaptic currents), as well as on neuronal signalling (i.e., increased efficacy of synaptic responses, even for neurons located several cell layers away).

**NDs:** ND particles and thin-films have found applications in neurosciences as substrates for neuronal growth [125–127] and as microelectrode material [128–129], by virtue of their peculiar electrical and chemical properties and stability. Specht et al. [125] were among the first to report on the suitability of ND as a substrate material. By micro-contact printing, they deposited laminin on NDs to form patterned microstructures, on which they then observed the ordered growth of primary cortical neurons.

**Figure 4:**
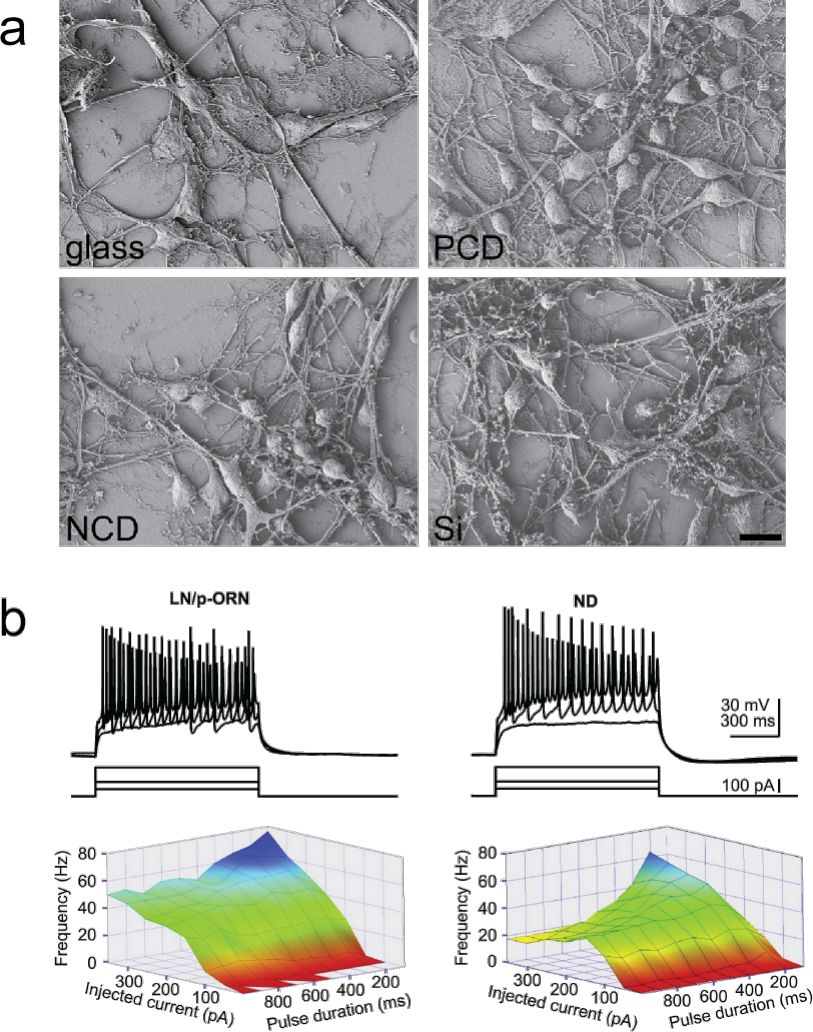
Scanning electron micrographs show that, when exposed to animal blood serum proteins, polycrystalline (PCD) and nanocrystalline diamond (NCD) substrates performed as good as glass or silicon substrates (a). NDs coating did not alter single-cell excitability (b). Reproduced and modified from [130] with permission. Copyright 2010 Elsevier.

Neuronal adhesion and cell excitability have been studied by Ariano and co-workers [126] and by Thalhammer and colleagues [130], who observed that oxygen functionalisation [126] and deposition of a layer of 5–10 nm sized ND particles (NDPs) [130] (Figure 4a) improved cell attachment and did not compromise neuronal electrical activity. This has been quantified by patch-clamp electrophysiology in terms of activation of voltage-gated Ca^2+^ channels [126] and of AP firing frequency [130] (Figure 4b). In the same researches, synaptic connectivity was also studied, by evoking inhibitory GABAergic responses [126] and by monitoring the spontaneous excitatory postsynaptic miniature currents (mEPSCs) [130]. The results showed that culturing neurons on ND did not affect synaptic activity.

The use of ND as a platform material for neural interfaces [131] has been studied both in vivo [132], ex vivo [133] and in vitro [128]. For these applications, boron doping of ND [134–135] has often been considered to bestow ND metallic properties, thus enabling superior S/N performances in the detection of neuronal activity and a wider electrochemical window for electrical stimulation.

**Figure 5:**
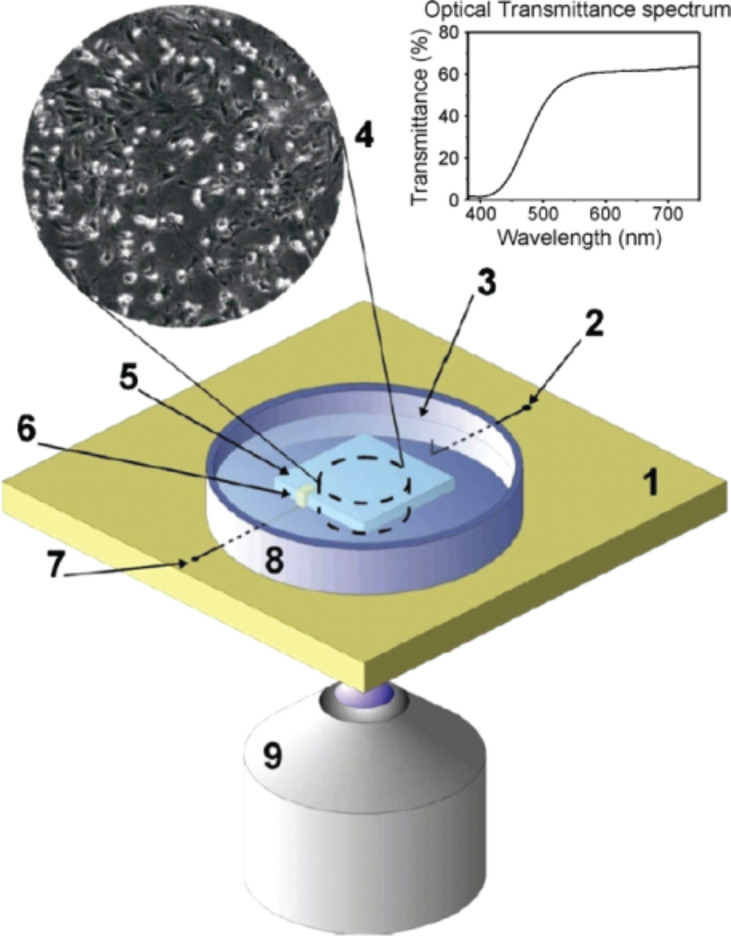
Sketch of the device for recording the extracellular electrical activity of cultured neuronal networks, developed by Ariano and co-workers. Cells (4) are plated in the recording chamber (8), directly onto hydrogen-terminated diamond (5). Reproduced from [128] with permission. Copyright 2009 Elsevier.

Ariano et al. [128] developed a ND-based device in order to record the spontaneous extracellular electrical activity in a murine neuronal cell line, which yielded results in good agreement with recordings made by means of conventional MEAs (Figure 5).

Single-spin NV-NDs embedded in an artificial lipid bilayer [136] and in a real cell membrane, in which there is a notable potential drop between the extra- and intracellular compartment, can be used as nanoscopic magnetic sensors of spin labels. Considering the typical values of membrane thickness and membrane potential, the order of magnitude for the expected electric fields is 10^7^ V·m^−1^, which can allow for the measurement of membrane potentials over a millisecond time-scale in real time.

Hall et al. [137] recently proposed the use of a single crystal ND substrate containing a layer of NV as a non-invasive model for a detection set-up based on Förster resonance energy transfer and optically detected magnetic resonance.

**Graphene:** Researchers have only recently begun to investigate graphene and its derivatives as potential scaffolds for neuronal growth, and as a conductive polymer for bio-interfacing. The interest that graphene has attracted was the result not only of its excellent conductive properties, but also because its nanostructure and its chemical stability render it a good candidate for favouring cell adhesion.

In one of the first studies, Li and co-workers [138] studied the biocompatibility of graphene and the contingent changes in the expression of the protein GAP43, which is associated with the growth of neurites. Their results showed high biocompatibility, and highlighted a longer average length of neurites, as well as better viability for cells grown on graphene, when compared to polystyrene control substrates. The authors also found an overexpression of the GAP43 protein, possibly the result of improved neurites outgrowth due to both the high electrical conductivity of graphene (as in CNTs) and by the wrinkled nanoscale morphology of graphene layer. These properties render graphene a good adhesion substrate to cells.

The use of graphene as an in vitro or an in vivo stimulator device was the primary focus of a study conducted by Heo and colleagues [95], who developed a graphene/PET film to test the effects of a non-contact field stimulation on cell-to-cell coupling. This film was found to be biocompatible and improved cell proliferation and viability compared to those observed in the control cultures; additionally, the electrical stimulation resulted in affecting the regulation of cytoskeleton protein related to cellular mobility, such as actin, resulting in morphological changes in cellular edges.

Sahni et al. [139] compared the neurite outgrowth of rat primary cortical neurons cultured on bare, graphene- and poly-D-lysine (PDL)-coated plastic polymer dishes, and found that neuronal viability showed remarkable differences between graphene and PDL substrates; the morphology of cells cultured on graphene displayed more linear dendritic structures compared to cells cultured on PDL and in control conditions. The improved neuronal adhesion on graphene, compared to the bare plastic polymeric dish, was ascribed by the authors to van der Waals forces between cell membranes and graphene.

Similarly to NDs [140], surface charges can influence adhesion and outgrowth of neuronal cells on graphene substrates. This aspect has been investigated by Tu and colleagues [141], who cultured primary rat hippocampal neurons on carboxylated GO (GO-COOH; negative surface charge; control condition), the surface of which had been chemically modified by functionalisation with three functional groups: methoxy (-OCH_3_; almost neutral surface charge), amino (NH_2_; positively charged surface) and poly-*m*-aminobenzene sulfonic acid (-NH_2_/-SO_3_H, PABS; zwitterionic). The viability of neurons after 7 DIV was estimated to be over 90% and, although no relevant differences in morphology were observed, neurons cultured on a positively charged surface showed a greater number of neurites per neuron, a longer length of neurites and a greater number of branches per neurite. However, the lack of a direct comparison with the conventional control conditions (i.e., glass or plastic, polymeric, culture substrates) makes it difficult to interpret these results in terms of a possible application for neural stimulation.

Graphene-based substrates have also been investigated as scaffolds for growth and for the differentiation of stem cells [142–143]. The differentiation into neurons of human neural stem cells (hNSCs), cultured on graphene has been studied by Park et al. [144]; the results showed not only an improved differentiation but also an enhanced cell adhesion and neurites formation compared to control conditions. Moreover, the expression of laminin-related receptors and of genes involved in the calcium signalling pathway was up-regulated for hNSCs grown on graphene.

Akhavan et al. [145] studied the differentiation of hNSCs on GO nanogrids deposited on a substrate made of TiO_2_ nanoparticles over SiO_2_, which made them photosensitive. The authors observed an increase in cell growth and alignment along the geometrical pattern of the nanogrids, further enhanced by means of a repeated photo stimulation.

**Figure 6:**
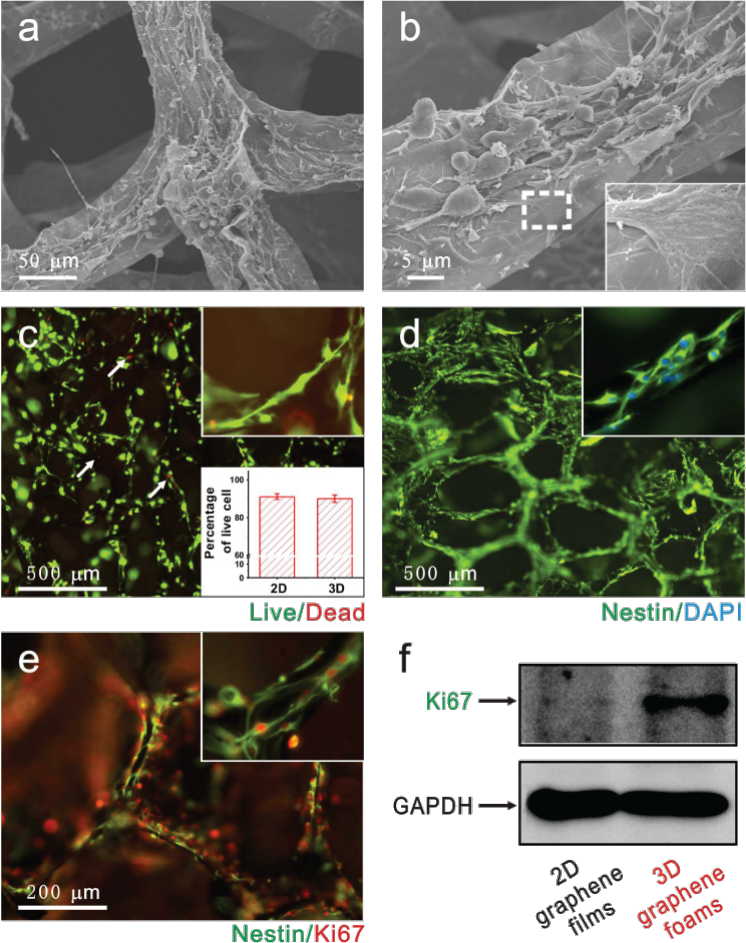
Three-dimensional graphene foam scaffolds allow neural stem cells to adhere and improve their proliferation by up-regulating Ki-67 protein expression. Reproduced from [146] with permission. Copyright 2013 Nature Publishing Group.

Li and co-workers [146] designed a three-dimensional graphene foam scaffold for neural stem cells. This scaffold allowed the formation of a three-dimensional neural network, resulting in an excellent substrate for cell adhesion and proliferation by up-regulating Ki-67 protein expression (Figure 6). To test whether such a scaffold could be used as a neural stimulation electrode, its electrochemical properties were investigated by cyclic voltammetry, the results showing that electrical stimulation via a capacitive charge injection was enhanced compared to the use of conventional graphene film electrode; this was due to the larger specific surface area of the three-dimensional scaffold.

## Conclusion

In this review, several reasons were established for why carbon-based nanomaterials have gained more importance over the past few years in the context of biomedical applications. We reviewed their most important applications, with an emphasis on the field of neuroscience and underlined their unique affinity for neuronal interfacing.

Ultimately, additional research is required before a complete understanding can be reached concerning how to best engineer these nanomaterials for their use in advanced applications in the fields of neuroscience and neuroprosthetics, as it relates to biocompatibility, biodegradability, biostability and the exact interaction mechanisms between these nanomaterials and neuronal networks. The most recent results in this field, however, are extremely promising and confirm the start of an important new discipline at the interface between nanotechnologies and neuroscience.

## References

[1] Iijima S (1991). Nature.

[2] Dresselhaus M S, Dresselhaus G, Avouris P (2001). Carbon Nanotubes Synthesis, Structure, Properties, and Applications.

[3] Wilder J W G, Venema L C, Rinzler A G, Smalley R E, Dekker C (1998). Nature.

[4] Charlier J-C, Blase X, Roche S (2007). Rev Mod Phys.

[5] Shtogun Y V, Woods L M (2009). J Phys Chem C.

[6] Oliva-Avilés A I, Avilés F, Sosa V, Oliva A I, Gamboa F (2012). Nanotechnology.

[7] Falvo M R, Clary G J, Taylor R M, Chi V, Brooks F P, Washburn S, Superfine R (1997). Nature.

[8] Field J E (1992). The Properties of natural and synthetic diamond.

[9] Liu H D, Dandy D S (1995). Diamond chemical vapor deposition: Nucleation and Early Growth Stages.

[10] Kulakova I I (2004). Phys Solid State.

[11] Dement’ev A P, Maslakov K I (2004). Phys Solid State.

[12] Palosz B, Pantea C, Grzanka E, Stelmakh S, Proffen Th, Zerda T W, Palosz W (2006). Diamond Relat Mater.

[13] Krüger A, Kataoka F, Ozawa M, Fujino T, Suzuki Y, Aleksenskii A E, Vul’ A Ya, Ōsawa E (2005). Carbon.

[14] Krüger A, Liang Y, Jarre G, Stegk J (2006). J Mater Chem.

[15] Aharonovich I, Castelletto S, Simpson D A, Su C-H, Greentree A D, Prawer S (2011). Rep Prog Phys.

[16] Aleksenskii A E, Osipov V Yu, Vul’ A Ya, Ber B Ya, Smirnov A B, Melekhin V G, Adriaenssens G J, Iakoubovskii K (2001). Phys Solid State.

[17] Kompan M E, Terukov E I, Gordeev S K, Zhukov S G, Nikolaev Yu A (1997). Phys Solid State.

[18] Doherty M W, Manson N B, Delaney P, Jelezko F, Wrachtrup J, Hollenberg L C L (2013). Phys Rep.

[19] Lee S-Y, Widmann M, Rendler T, Doherty M W, Babinec T M, Yang S, Eyer M, Siyushev P, Hausmann B J M, Loncar M (2013). Nat Nanotechnol.

[20] Wrachtrup J, Jelezko F (2006). J Phys: Condens Matter.

[21] Tisler J, Balasubramanian G, Naydenov B, Kolesov R, Grotz B, Reuter R, Boudou J-P, Curmi P A, Sennour M, Thorel A (2009). ACS Nano.

[22] Novoselov K S, Geim A K, Morozov S V, Jiang D, Zhang Y, Dubonos S V, Grigorieva I V, Firsov A A (2004). Science.

[23] Peierls R E (1935). Ann Inst Henri Poincare.

[24] Mermin N D (1968). Phys Rev.

[25] Novoselov K S, Jiang Z, Zhang Y, Morozov S V, Stormer H L, Zeitler U, Maan J C, Boebinger G S, Kim P, Geim A K (2007). Science.

[26] Bolotin K I, Sikes K J, Jiang Z, Klima M, Fudenberg G, Hone J, Kim P, Stormer H L (2008). Solid State Commun.

[27] Novoselov K S, Jiang D, Schedin F, Booth T J, Khotkevich V V, Morozov S V, Geim A K (2005). Proc Natl Acad Sci U S A.

[28] Katsnelson M I, Novoselov K S, Geim A K (2006). Nat Phys.

[29] Lee C, Wei X, Kysar J W, Hone J (2008). Science.

[30] Nair R R, Blake P, Grigorenko A N, Novoselov K S, Booth T J, Stauber T, Peres N M R, Geim A K (2008). Science.

[31] Stoller M D, Park S, Zhu Y, An J, Ruoff R S (2008). Nano Lett.

[32] Yang Z, Zhang Y, Yang Y, Sun L, Han D, Li H, Wang C (2010). Nanomedicine.

[33] Flavin K, Kopf I, Del Canto E, Navio C, Bittencourt C, Giordani S (2011). J Mater Chem.

[34] Georgakilas V, Kordatos K, Prato M, Guldi D M, Holzinger M, Hirsch A (2002). J Am Chem Soc.

[35] Richard C, Balavoine F, Schultz P, Ebbesen T W, Mioskowski C (2003). Science.

[36] Zheng M, Jagota A, Semke E D, Diner B A, McLean R S, Lustig S R, Richardson R E, Tassi N G (2003). Nat Mater.

[37] Dyke C A, Tour J M (2004). Chem – Eur J.

[38] Dyke C A, Tour J M (2004). J Phys Chem A.

[39] Tagmatarchis N, Prato M (2004). J Mater Chem.

[40] Khabashesku V N (2011). Russ Chem Rev.

[41] Coleman K S, Bailey S R, Fogden S, Green M L H (2003). J Am Chem Soc.

[42] Liang F, Sadana A K, Peera A, Chattopadhyay J, Gu Z, Hauge R H, Billups W E (2004). Nano Lett.

[43] Yang M, Flavin K, Kopf I, Radics G, Hearnden C H, McManus G J, Moran B, Villalta-Cerdas A, Echegoyen L A, Giordani S (2013). Small.

[44] Porter A E, Gass M, Muller K, Skepper J N, Midgley P A, Welland M (2007). Nat Nanotechnol.

[45] Shvedova A A, Kisin E R, Mercer R, Murray A R, Johnson V J, Potapovich A I, Tyurina Y Y, Gorelik O, Arepalli S, Schwegler-Berry D (2005). Am J Physiol: Lung Cell Mol Physiol.

[46] Poland C A, Duffin R, Kinloch I, Maynard A, Wallace W A H, Seaton A, Stone V, Brown S, Macnee W, Donaldson K (2008). Nat Nanotechnol.

[47] Bottini M, Bruckner S, Nika K, Bottini N, Bellucci S, Magrini A, Bergamaschi A, Mustelin T (2006). Toxicol Lett.

[48] Ding L, Stilwell J, Zhang T, Elboudwarej O, Jiang H, Selegue J P, Cooke P A, Gray J W, Chen F F (2005). Nano Lett.

[49] Dumortier H, Lacotte S, Pastorin G, Marega R, Wu W, Bonifazi D, Briand J P, Prato M, Muller S, Bianco A (2006). Nano Lett.

[50] Yehia H N, Draper R K, Mikoryak C, Walker E K, Bajaj P, Musselman I H, Daigrepont M C, Dieckmann G R, Pantano P (2007). J Nanobiotechnol.

[51] Wu W, Li R, Bian X, Zhu Z, Ding D, Li X, Jia Z, Jiang X, Hu Y (2009). ACS Nano.

[52] Pantarotto D, Singh R, McCarthy D, Erhardt M, Briand J-P, Prato M, Kostarelos K, Bianco A (2004). Angew Chem.

[53] Gao L, Nie L, Wang T, Qin Y, Guo Z, Yang D, Yan X (2006). ChemBioChem.

[54] Cella L N, Chen W, Myung N V, Mulchandani A (2010). J Am Chem Soc.

[55] Madani S Y, Naderi N, Dissanayake O, Tan A, Seifalian A M (2011). Int J Nanomed.

[56] Elhissi A M A, Ahmed W, Ul Hassan I, Dhanak V R, D'Emanuele A (2012). J Drug Delivery.

[57] Hirata E, Uo M, Takita H, Akasaka T, Watari F, Yokoyama A (2011). Carbon.

[58] Zavaleta C, de la Zerda A, Liu Z, Keren S, Cheng Z, Schipper M, Chen X, Dai H, Gambhir S S (2008). Nano Lett.

[59] Jin H, Heller D A, Strano M S (2008). Nano Lett.

[60] Sucapane A, Cellot G, Prato M, Giugliano M, Parpura V, Ballerini L (2009). J Nanoneurosci.

[61] Chan V, Raman R, Cvetkovic C, Bashir R (2013). ACS Nano.

[62] Martinelli V, Cellot G, Toma F M, Long C S, Caldwell J H, Zentilin L, Giacca M, Turco A, Prato M, Ballerini L (2012). Nano Lett.

[63] Martinelli V, Cellot G, Toma F M, Long C S, Caldwell J H, Zentilin L, Giacca M, Turco A, Prato M, Ballerini L (2013). ACS Nano.

[64] Lin Z Q, Liu L H, Xi Z G, Huang J H, Lin B C (2012). Int J Nanomed.

[65] Huang H, Pierstorff E, Ōsawa E, Ho D (2007). Nano Lett.

[66] Li J, Zhu Y, Li W, Zhang X, Peng Y, Huang Q (2010). Biomaterials.

[67] Metzler P, von Wilmowsky C, Stadlinger B, Zemann W, Schlegel K A, Rosiwal S, Rupprecht S (2013). J Cranio-Maxillofacial Surg.

[68] Chang I P, Hwang K C, Chiang C-S (2008). J Am Chem Soc.

[69] Yu S-J, Kang M-W, Chang H-C, Chen K-M, Yu Y-C (2005). J Am Chem Soc.

[70] Schrand A M, Dai L, Schlager J J, Hussain S M, Ōsawa E (2007). Diamond Relat Mater.

[71] Schrand A M, Huang H, Carlson C, Schlager J J, Omacr Sawa E, Hussain S M, Dai L (2007). J Phys Chem B.

[72] Schrand A M, Lin J B, Shenderova O A, Gruen D M (2012). Characterization of Detonation Nanodiamonds. Ultrananocrystalline Diamond.

[73] Bakowicz-Mitura K, Bartosz G, Mitura S (2007). Surf Coat Technol.

[74] Xing Y, Xiong W, Zhu L, Ōsawa E, Hussin S, Dai L (2011). ACS Nano.

[75] Vaijayanthimala V, Tzeng Y-K, Chang H-C, Li C-L (2009). Nanotechnology.

[76] Schrand A M, Lin J B, Hens S C, Hussain S M (2011). Nanoscale.

[77] Yuan Y, Wang X, Jia G, Liu J-H, Wang T, Gu Y, Yang S-T, Zhen S, Wang H, Liu Y (2010). Diamond Relat Mater.

[78] Puzyr A P, Baron A V, Purtov K V, Bortnikov E V, Skobelev N N, Mogilnaya O A, Bondar V S (2007). Diamond Relat Mater.

[79] Yuan Y, Chen Y, Liu J-H, Wang H, Liu Y (2009). Diamond Relat Mater.

[80] Rojas S, Gispert J D, Martín R, Abad S, Menchón C, Pareto D, Victor V M, Álvaro M, García H, Herance J R (2011). ACS Nano.

[81] Lam R, Chen M, Pierstorff E, Huang H, Ōsawa E, Ho D (2008). ACS Nano.

[82] Chen M, Pierstorff E D, Lam R, Li S Y, Huang H, Ōsawa E, Ho D (2009). ACS Nano.

[83] Adnan A, Lam R, Chen H, Lee J, Schaffer D J, Barnard A S, Schatz G C, Ho D, Liu W K (2011). Mol Pharmaceutics.

[84] Li Y, Zhou X, Wang D, Yang B, Yang P (2011). J Mater Chem.

[85] Bondar’ V S, Pozdnyakova I O, Puzyr’ A P (2004). Phys Solid State.

[86] Kong X L, Huang L C L, Hsu C-M, Chen W-H, Han C-C, Chang H-C (2005). Anal Chem.

[87] Akhavan O, Ghaderi E, Akhavan A (2012). Biomaterials.

[88] Chang Y, Yang S-T, Liu J-H, Dong E, Wang Y, Cao A, Liu Y, Wang H (2011). Toxicol Lett.

[89] Yuan J, Gao H, Sui J, Duan H, Chen W N, Ching C B (2012). Toxicol Sci.

[90] Yue H, Wei W, Yue Z, Wang B, Luo N, Gao Y, Ma D, Ma G, Su Z (2012). Biomaterials.

[91] Feng L, Zhang S, Liu Z (2011). Nanoscale.

[92] Sun X, Liu Z, Welsher K, Robinson J T, Goodwin A, Zaric S, Dai H (2008). Nano Res.

[93] Hong H, Yang K, Zhang Y, Engle J W, Feng L, Yang Y, Nayak T R, Goel S, Bean J, Theuer C P (2012). ACS Nano.

[94] Kalbacova M, Broz A, Kong J, Kalbac M (2010). Carbon.

[95] Heo C, Yoo J, Lee S, Jo A, Jung S, Yoo H, Lee Y H, Suh M (2011). Biomaterials.

[96] Yang K, Zhang S, Zhang G, Sun X, Lee S-T, Liu Z (2010). Nano Lett.

[97] Yang K, Wan J, Zhang S, Tian B, Zhang Y, Liu Z (2012). Biomaterials.

[98] Dey R S, Raj C R (2010). J Phys Chem C.

[99] Tang L, Wang Y, Li Y, Feng H, Lu J, Li J (2009). Adv Funct Mater.

[100] Kim Y R, Bong S, Kang Y J, Yang Y, Mahajan R K, Kim J S, Kim H (2010). Biosens Bioelectron.

[101] Sun C-L, Lee H-H, Yang J-M, Wu C-C (2011). Biosens Bioelectron.

[102] Yang X, Zhang X, Liu Z, Ma Y, Huang Y, Chen Y (2008). J Phys Chem C.

[103] Zhang L, Xia J, Zhao Q, Liu L, Zhang Z (2010). Small.

[104] Weaver C L, Larosa J M, Luo X, Cui X T (2014). ACS Nano.

[105] Mattson M P, Haddon R C, Rao A M (2000). J Mol Neurosci.

[106] Voge C M, Stegemann J P (2011). J Neural Eng.

[107] Malarkey E B, Parpura V (2007). Neurodegener Dis.

[108] Ben-Jacob E, Hanein Y (2008). J Mater Chem.

[109] Keefer E W, Botterman B R, Romero M I, Rossi A F, Gross G W (2008). Nat Nanotechnol.

[110] Malarkey E B, Fisher K A, Bekyarova E, Liu W, Haddon R C, Parpura V (2009). Nano Lett.

[111] Gabay T, Ben-David M, Kalifa I, Sorkin R, Abrams Z R, Ben-Jacob E, Hanein Y (2007). Nanotechnology.

[112] Gabriel G, Gómez R, Bongard M, Benito N, Fernández E, Villa R (2009). Biosens Bioelectron.

[113] Shein M, Greenbaum A, Gabay T, Sorkin R, David-Pur M, Ben-Jacob E, Hanein Y (2009). Biomed Microdevices.

[114] Fuchsberger K, Le Goff A, Gambazzi L, Toma F M, Goldoni A, Giugliano M, Stelzle M, Prato M (2011). Small.

[115] Gerwig R, Fuchsberger K, Schroeppel B, Link G S, Heusel G, Kraushaar U, Schuhmann W, Stett A, Stelzle M (2012). Front Neuroeng.

[116] Lin C-M, Lee Y-T, Yeh S-R, Fang W (2009). Biosens Bioelectron.

[117] David-Pur M, Bareket-Keren L, Beit-Yaakov G, Raz-Prag D, Hanein Y (2014). Biomed Microdevices.

[118] Lovat V, Pantarotto D, Lagostena L, Cacciari B, Grandolfo M, Righi M, Spalluto G, Prato M, Ballerini L (2005). Nano Lett.

[119] Mazzatenta A, Giugliano M, Campidelli S, Gambazzi L, Businaro L, Markram H, Prato M, Ballerini L (2007). J Neurosci.

[120] Sorkin R, Greenbaum A, David-Pur M, Anava S, Ayali A, Ben-Jacob E, Hanein Y (2009). Nanotechnology.

[121] Cellot G, Cilia E, Cipollone S, Rancic V, Sucapane A, Giordani S, Gambazzi L, Markram H, Grandolfo M, Scaini D (2009). Nat Nanotechnol.

[122] Fabbro A, Sucapane A, Toma F M, Calura E, Rizzetto L, Carrieri C, Roncaglia P, Martinelli V, Scaini D, Masten L (2013). PLoS One.

[123] Cellot G, Toma F M, Varley Z K, Laishram J, Villari A, Quintana M, Cipollone S, Prato M, Ballerini L (2011). J Neurosci.

[124] Fabbro A, Villari A, Laishram J, Scaini D, Toma F M, Turco A, Prato M, Ballerini L (2012). ACS Nano.

[125] Specht C G, Williams O A, Jackman R B, Schoepfer R (2004). Biomaterials.

[126] Ariano P, Baldelli P, Carbone E, Gilardino A, Lo Giudice A, Lovisolo D, Manfredotti C, Novara M, Sternschulte H, Vittone E (2005). Diamond Relat Mater.

[127] May P W, Regan E M, Taylor A, Uney J, Dick A D, McGeehan J (2012). Diamond Relat Mater.

[128] Ariano P, Lo Giudice A, Marcantoni A, Vittone E, Carbone E, Lovisolo D (2009). Biosens Bioelectron.

[129] Ho-Yin C, Aslam D M, Wiler J A, Casey B (2009). J Microelectromech Syst.

[130] Thalhammer A, Edgington R J, Cingolani L A, Schoepfer R, Jackman R B (2010). Biomaterials.

[131] Garrett D J, Ganesan K, Stacey A, Fox K, Meffin H, Prawer S (2012). J Neural Eng.

[132] Halpern J M, Cullins M J, Chiel H J, Martin H B (2010). Diamond Relat Mater.

[133] Bonnauron M, Saada S, Mer C, Gesset C, Williams O A, Rousseau L, Scorsone E, Mailley P, Nesladek M, Arnault J-C (2008). Phys Status Solidi A.

[134] Feoktistov N A, Grudinkin S A, Rybin M V, Smirnov A N, Aleksenskii A E, Vul’ A Y, Golubev V G (2011). Tech Phys Lett.

[135] Bennet K, Lee K, Kruchowski J, Chang S-Y, Marsh M, Van Orsow A, Paez A, Manciu F (2013). Materials.

[136] Kaufmann S, Simpson D A, Hall L T, Perunicic V, Senn P, Steinert S, McGuinness L P, Johnson B C, Ohshima T, Caruso F (2013). Proc Natl Acad Sci U S A.

[137] Hall L T, Beart G C C, Thomas E A, Simpson D A, McGuinness L P, Cole J H, Manton J H, Scholten R E, Jelezko F, Wrachtrup J (2012). Sci Rep.

[138] Li N, Zhang X, Song Q, Su R, Zhang Q, Kong T, Liu L, Jin G, Tang M, Cheng G (2011). Biomaterials.

[139] Sahni D, Jea A, Mata J A, Marcano D C, Sivaganesan A, Berlin J M, Tatsui C E, Sun Z, Luerssen T G, Meng S (2013). J Neurosurg: Pediatr.

[140] Lechleitner T, Klauser F, Seppi T, Lechner J, Jennings P, Perco P, Mayer B, Steinmuller-Nethl D, Preiner J, Hinterdorfer P (2008). Biomaterials.

[141] Tu Q, Pang L, Chen Y, Zhang Y, Zhang R, Lu B, Wang J (2014). Analyst.

[142] Nayak T R, Andersen H, Makam V S, Khaw C, Bae S, Xu X, Ee P-L R, Ahn J-H, Hong B H, Pastorin G (2011). ACS Nano.

[143] Lee W C, Lim C H Y X, Shi H, Tang L A L, Wang Y, Lim C T, Loh K P (2011). ACS Nano.

[144] Park S Y, Park J, Sim S H, Sung M G, Kim K S, Hong B H, Hong S (2011). Adv Mater.

[145] Akhavan O, Ghaderi E (2013). J Mater Chem B.

[146] Li N, Zhang Q, Gao S, Song Q, Huang R, Wang L, Liu L, Dai J, Tang M, Cheng G (2013). Sci Rep.

