# Correction: Carbon-based smart nanomaterials in biomedicine and neuroengineering

**DOI:** 10.3762/bjnano.6.51

**Published:** 2015-02-18

**Authors:** Antonina M Monaco, Michele Giugliano

**Affiliations:** 1 Theoretical Neurobiology and Neuroengineering Lab, Department of Biomedical Sciences, University of Antwerp, Universiteitsplein 1, B-2610 Wilrijk, Belgium; 2 Brain Mind Institute, Swiss Federal Institute of Technology, Lausanne, CH-1015, Switzerland; 3 Department of Computer Science, University of Sheffield, S1 4DP Sheffield, UK

**Keywords:** carbon nanotubes, electrophysiology, graphene, microelectrodes, nanodiamonds, nanotechnology, neuroengineering, neuronal cultures, neuroscience

Correction for the Acknowledgement section, the correct text should be:

## Acknowledgements

We are grateful to Drs. C. Bittencourt, M. Prato, L. Ballerini, and M. Nesládek for discussions. We are also grateful to Drs. Henry Markram, Sébastien Lasserre, and the Blue Brain Project team at the EPFL for contributing the graphical abstract. Financial support from the European Commission (FP7-PEOPLE-IEF “INCA-NANEP”, contract n. 328214; FP7-NMP "MERIDIAN" project, contract n. 280778-02), from the Flanders Research Foundation (contract no. G088812N), and from the Belgian Science Policy Office (BELSPO) is kindly acknowledged.

